# Studies of Zinc(II) and Nickel(II) Complexes of GSH, GSSG and Their Analogs Shed More Light on Their Biological Relevance

**DOI:** 10.1155/S1565363304000172

**Published:** 2004

**Authors:** Artur Krężel, Wojciech Bal

**Affiliations:** 1 Preventive Medicine and Community Health University of Texas Medical Branch 700 Harborside Drive Ewing Hall Galveston Island TX 77555 USA; 2 Institute of Biochemistry and Biophysics Polish Academy of Sciences Pawińskiego 5a Warsaw 02-106 Poland

## Abstract

Glutathione, Υ-Glu-Cys-Gly, is one of the most abundant small molecules in biosphere. Its main form is the reduced monomer (GSH), serving to detoxicate xenobiotics and heavy metals, reduce protein thiols, maintain cellular membranes and deactivate free radicals. Its oxidized dimer (GSSG) controls metal content of metallothionein. The results presented provided a quantitative and structural description of Zn(II)- glutathione complexes, including a novel ternary Zn(II)-GSH-His complex. A solution structure for this complex was obtained using 2D-NMR. The Complexes studied may contribute to both zinc and glutathione physiology. In the case of Ni(ll) complexes an interesting dependence of coordination modes on the ratios of reactants was found. At high GSH excess a Ni(GSH)2 complex is formed, with Ni(ll) bonded through S and N and/or O donor atoms. This complex may exist as a high- or low-spin species. Another goal of the studies presented was to describe the catalytic properties of Ni(II) ions towards GSH oxidation, which appeared to be an important step in nickel carcinogenesis. The pH dependence of oxidation rates allowed to determine the Ni(GSH)_2_ complex as the most active among the toxicologically relevant species. Protonation and oxidation of metal-free GSH and its analogues were also studied in detail. The monoprotonated form HL^2-^ of GSH is the one most susceptible to oxidation, due to a salt bridge between S^-^ and NH_3_^+^ groups, which activates the thiol.


### Studies of Zinc(II) and Nickel(II) Complexes of GSH, GSSG and Their Analogs Shed More Light on Their Biological Relevance

Artur Krężel[a,*] and Wojciech Bal[b,‡]

*[a]Preventive Medicine and Community Health, University of Texas Medical Branch, 700 Harborside Drive, Ewing Hall, Galveston Island, TX 77555 USA*
*e-mail: arkrezel@utmb.edu*
*[b]Institute of Biochemistry and Biophysics, Polish Academy of Sciences, Pawińskiego 5a, 02-106 Warsaw, Poland, e-mail: wbal@ibb.waw.pl.*

**ABSTRACT**

Glutathione, γ-Glu-Cys-Gly, is one of the most abundant small molecules in biosphere. Its main form is the reduced monomer (GSH), serving to detoxicate xenobiotics and heavy metals, reduce protein thiols, maintain cellular membranes and deactivate free radicals. Its oxidized dimer (GSSG) controls metal content of metallothionein. The results presented provided a quantitative and structural description of Zn(II)-glutathione complexes, including a novel ternary Zn(II)-GSH-His complex. A solution structure for this complex was obtained using 2D-NMR. The complexes studied may contribute to both zinc and glutathione physiology. In the case of Ni(II) complexes an interesting dependence of coordination modes on the ratios of reactants was found. At high GSH excess a $Ni(GSH)_2$ complex is formed, with Ni(II) bonded through S and N and/or O donor atoms. This complex may exist as a high- or low-spin species. Another goal of the studies presented was to describe the catalytic properties of Ni(II) ions towards GSH oxidation, which appeared to be an important step in nickel carcinogenesis. The pH dependence of oxidation rates allowed to determine the $Ni(GSH)_2$ complex as the most active among the toxicologically relevant species. Protonation and oxidation of metal-free GSH and its analogues were also studied in detail. The monoprotonated form $HL^{2-}$ of GSH is the one most susceptible to oxidation, due to a salt bridge between $S^-$ and $NH_3^+$ groups, which activates the thiol.

### INTRODUCTION

Reduced glutathione (GSH) is a non-protein tripeptide of the sequence γ-Glu-Cys-Gly. It is present in biological fluids and serves a variety of fundamental physiological functions. The intracellular concentration of GSH in human cells is often as high as 1 - 20 mM. This makes it one of the most important organic

---

* To whom correspondence should be addressed.

‡ Polish Academy of Sciences substances present in the human body /1, 2/. The most important functions of glutathione include detoxication of xenobiotics and heavy metals, reduction of oxidation-prone protein thiols, maintenance of cellular membranes and deactivation of free radicals /3/. Its disulfide, GSSG, restores disulfide bridges and co-regulates metal content of metallothionein /4, 5/.

Due to its intracellular abundance, GSH is a likely target for metal ions, especially those having high affinity for the thiolate sulfur /6, 7/. These metal ions include both biogenic elements, such as zinc, and xenobiotic toxins, such as nickel, whose intracellular transport remains to be elucidated.

Zinc is involved, among others, in DNA transcription (enzymes, zinc fingers) and intracellular signaling /8, 9/. It is known that GSSG releases Zn(II) from its storage protein, metallothionein /10, 11/. The transport of Zn(II) ions across the cellular membrane is mediated by dedicated protein shuttles /12, 13/. The mechanisms of Zn(II) delivery to its target proteins from these endpoints remain, however, unclear. One goal of our studies was therefore to provide chemical data that might help assessing the possible involvement of GSH/GSSG or other intracellular potential low molecular weight ligands in the cellular transport of Zn(II) ions.

Nickel compounds are human carcinogens. While it is agreed that intracellular Ni(II) is responsible for neoplastic transformation, several divergent concepts in nickel carcinogenesis have been developed, as reviewed recently /14/. Both direct genotoxic mechanisms, e.g. oxidative, and indirect ones, based on inhibition/destruction of proteins involved in the maintenance of genetic material, such as DNA repair systems, have gained support.

However, regardless of the actual mechanisms of nickel carcinogenesis, knowledge of the cellular speciation of Ni(II) is needed for the selection of feasible mechanisms. Also, previous studies indicated that the intracellular level of reduced glutathione (GSH) is an important factor in the process of cellular resistance to Ni(II), which, in turn, depletes cellular GSH stores /15-17/. Therefore, our second goal was to elucidate the equilibria and reactivities involving Ni(II) and GSH.

### ACID-BASE AND COORDINATION ASPECTS OF GSH AND GSSG

The molecule of GSH (Scheme 1A) possesses eight potential donors of electronic density toward metal ions. They can be grouped into three classes: the glutamic (amino acid-like) set of amine and carboxylate donors, the thiol, and the peptide bonds. The isolated carboxylate of glycine can be functionally included into the first class, but it often participates in metal coordination together with the thiol donor, due to the spatial constraints. GSSG (Scheme 1B), in place of the reactive and coordinationally attractive thiol group, contains a disulfide bridge, which interacts with metal ions very weakly /18/. Among these potential donors, the carboxyls, the amine and the thiol are protonated/deprotonated spontaneously in aqueous solution: GSH has a total of four such groups, and GSSG has a total of six, as shown on Scheme 1. At physiological conditions both GSH and GSSG exist as $H_2L^-$ ions, with deprotonated carboxylate groups and still protonated basic functions. The logarithmic values of protonation constants of GSH and GSSG are presented in Table 1.

A)

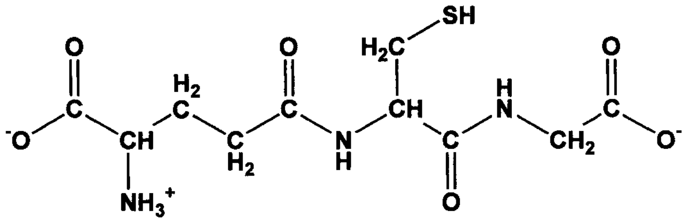

B)

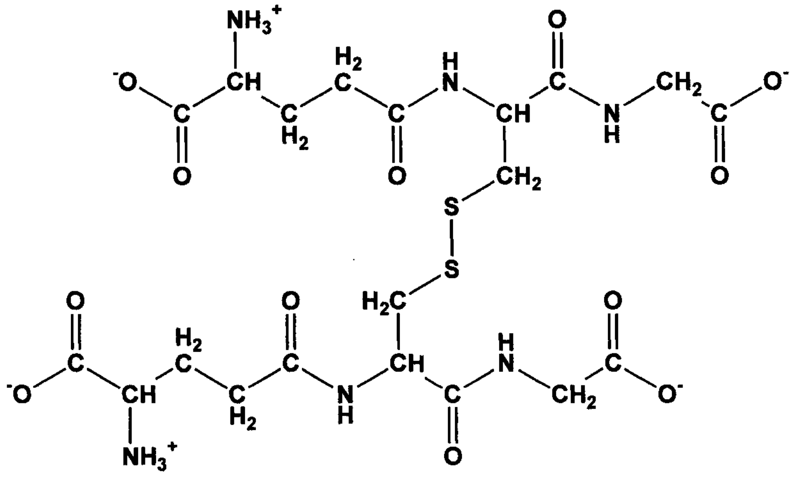

**Scheme 1.** The structures of reduced glutathione, GSH (A) and oxidized glutathione, GSSG (B) existing at physiological pH.

**Table 1**

Protonation constants of GSH and GSSG.

| Glutathione | Protonic species | log $\beta_{ijk}$ | p$K_a$ |
|---|---|---|---|
| GSH[a] | $HL^{2-}$ | 9.66 | 9.66 |
| | $H_2L^-$ | 18.39 | 8.74 |
| | $H_3L$ | 21.90 | 3.51 |
| | $H_4L^+$ | 24.03 | 2.13 |
| GSSG[b] | $HL^{3-}$ | 9.90 | 9.90 |
| | $H_2L^{2-}$ | 18.34 | 8.44 |
| | $H_3L^-$ | 22.16 | 3.82 |
| | $H_4L$ | 25.32 | 3.16 |
| | $H_5L^+$ | 27.71 | 2.39 |
| | $H_6L^{2+}$ | 29.50 | 1.79 |

[a] Ref. /25/.

[b] Ref. /20/.

### BINARY COMPLEXES OF ZINC(II) IONS WITH GLUTATHIONE

The Zn(II) ion, a borderline Lewis acid, is suited for interactions with such a versatile ligand as GSH. The simultaneous presence of aminoacid-like and thiolate functions in its molecule results in the variety of Zn(II) coordination modes, depending on pH and molar ratios. Studies of coordination equilibria performed by us and our predecessors, in particular those applying NMR techniques, demonstrated the involvement of the thiol function in Zn(II) coordination in the entire pH range of the binding /19-22/. The sulfur is accompanied by residues of Glu or Gly donors. At highly alkaline pH, the γ-Glu-Cys peptide nitrogen is deprotonated and coordinated to Zn(II) (Scheme 2). This manifests itself in negative indices at H atoms in respective stoichiometric formulae. Figure 1 presents an example of a species distribution for the Zn(II)/GSH system. The stability constants of complexes are presented in Table 2.

The absence of the thiol function in GSSG reduces the variety of complex forms markedly. The Glu residue is the main binding site for Zn(II), however, the ability of GSSG to form a 18-membered macrochelate ring and coordinate the Zn(II) ion with its both Glu residues results in a stability constant for its ML species almost as high as the one with GSH (Table 2).

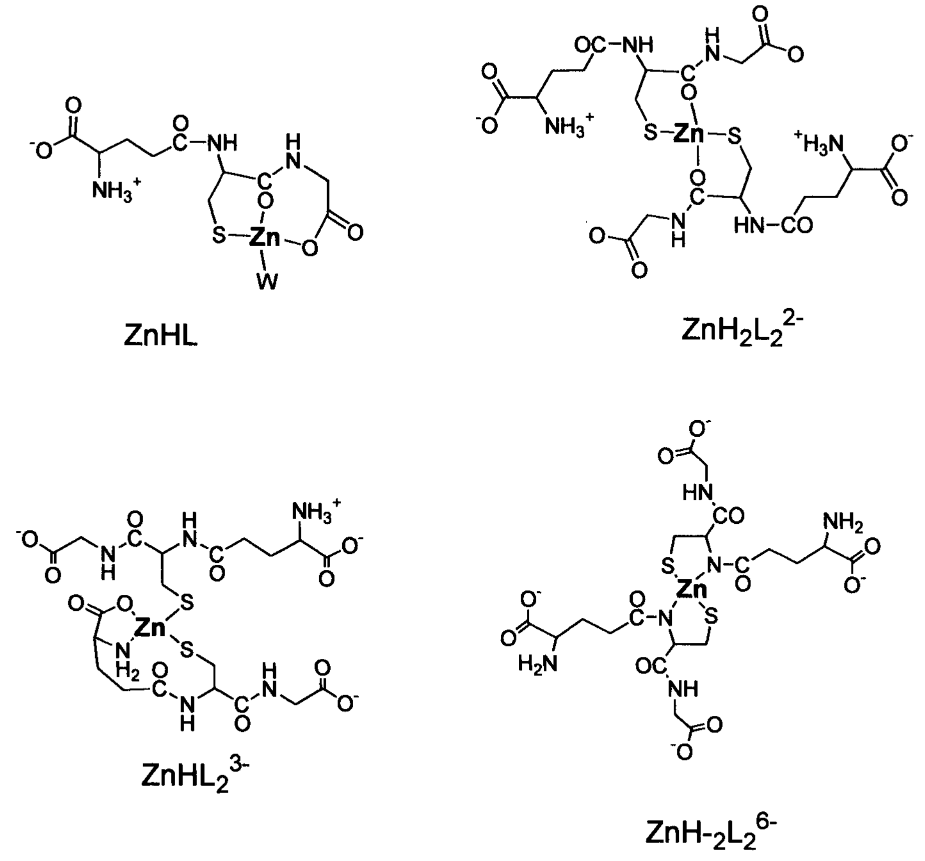

**Scheme 2.** The proposed structures of Zn(II)-GSH complexes. W denotes a water molecule.

**Table 2**

Stability constants of GSH and GSSG with Zn(II) and Ni(II)

| Glutathione | Complex Species | log $\beta_{ijk}$ | p$K_a$ |
|---|---|---|---|
| GSH[a,b] | ZnHL | 14.74 | - |
| | $ZnL^-$ | 8.31 | 6.43 |
| | $ZnH_2L_2^{2-}$ | 29.50 | - |
| | $ZnHL_2^{3-}$ | 22.533 | 6.97 |
| | $ZnL_2^{4-}$ | 13.617 | 8.92 |
| | $ZnH_{-1}L_2^{5-}$ | 3.817 | 9.80 |
| | $ZnH_{-2}L_2^{6-}$ | -6.485 | 10.28 |
| | NiHL | 14.70 | - |
| | $Ni_2L_2^{2-}$ | 17.81 | - |
| | $NiHL_2^{3-}$ | 20.40 | - |
| | $NiL_2^{4-}$ | 11.15 | 9.25 |
| | $NiH_{-1}L_2^{5-}$ | -0.05 | 11.20 |
| GSSG[c] | $ZnHL^-$ | 13.81 | - |
| | $ZnL^{2-}$ | 7.60 | 6.21 |
| | $Zn_2L$ | 9.8 | - |
| | $NiHL^-$ | 14.96 | - |
| | $NiL^{2-}$ | 9.08 | 5.88 |
| | $Ni_2L$ | 11.06 | - |

[a] Ref. /25/.

[b] Ref. /30/.

[c] Ref. /20/.

### TERNARY ZINC(II) COMPLEXES

The sterical constraints, due to the simultaneous coordination of the thiol and the glutamic acid donors, exclude the Gly carboxylate from the coordination to the same Zn(II) ion. As a result, the coordination sphere of the tetrahedral or five-coordinate Zn(II) (as common for thiol-containing complexes /23, 24/) cannot be saturated by one molecule of GSH. This feature makes the Zn(II)/GSH system prone for the formation of ternary complexes. The separation of the thiol from the Glu donors also facilitates the formation of monodentate, sulfur-only coordination mode. An example is provided in Scheme 3, where the structure of the ternary complex, containing GSH and L-His is presented, as obtained from 2D NMR studies /25/. This structure demonstrates the importance of various types of salt bridges and hydrogen bonds in the shaping of such ternary species.

A)

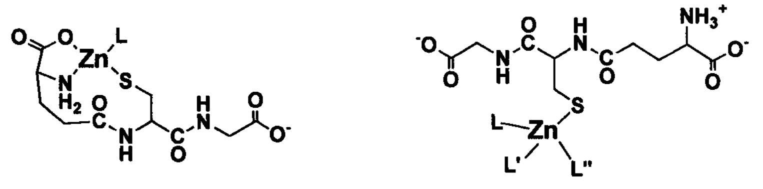

B)

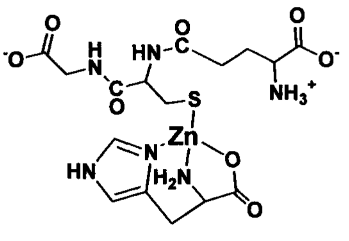

**Scheme 3.** Two motifs of zinc ternary complexes with glutathione (A), ternary complexes with L-His based on ref. /25/ (B). L, L' and L'' represents the other ligand or ligands in ternary complexes.

### COMPLEXES OF Ni(II) IONS WITH GLUTATHIONE

Nickel(II) exhibits little preference among sulfur, nitrogen and oxygen as potential donor atoms, similarly to zinc(II). This results in similarities in coordination modes for GSH between these two metal ions, limited however by differences in complex geometries, which are usually square-planar for thiolate-containing Ni(II) complexes and octahedral for the Ni(II) complexes without thiolate coordination /26/ (Figure 2). This difference results in the formation of oligomeric complexes at low GSH-to-Ni(II) ratios. In the conditions of GSH excess, which by the way are relevant physiologically, simpler monomeric or dimeric complexes are formed, stoichiometrically and structurally similar to those of Zn(II). They are presented in Scheme 4. Table 2 presents their stability constants, which are in a fair agreement with those determined previously /27, 28/. As seen in Scheme 4, all GSH complexes, except for NiHL, involve the thiolate coordination to Ni(II). In none of the cases the Gly carboxylate was identified as participating in Ni(II) binding. At high pH the γ-Glu-Cys peptide nitrogen is deprotonated and coordinated, in the $NiH_{-1}L_2^{5-}$ complex. Magnetic susceptibility measurements indicated that all complex species, except for the latter alkaline species, retain a substantial paramagnetism, which suggests the presence of an equilibrium between the high-spin and low-spin complexes /29, 30/.

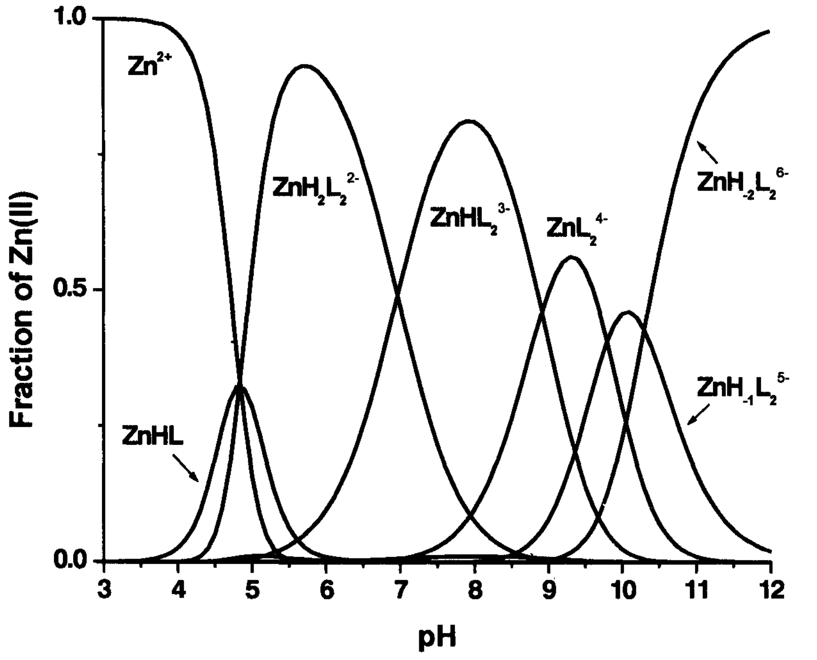

**Fig. 1:** The species distribution of Zn(II)-GSH complexes (4 mM GSH and 1 mM Zn(II)).

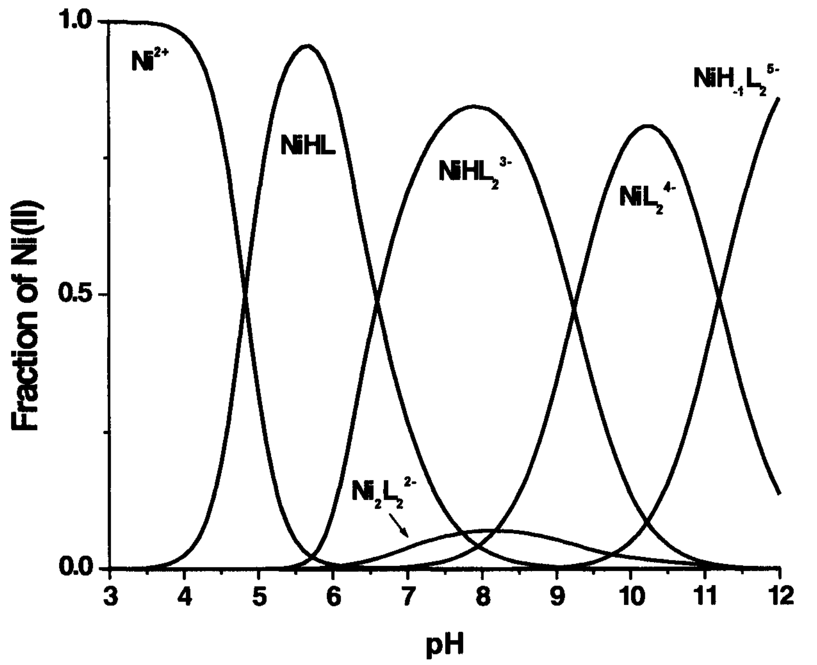

**Fig. 2:** The species distribution of Ni(II)-GSH complexes (4 mM GSH and 1 mM Ni(II)).

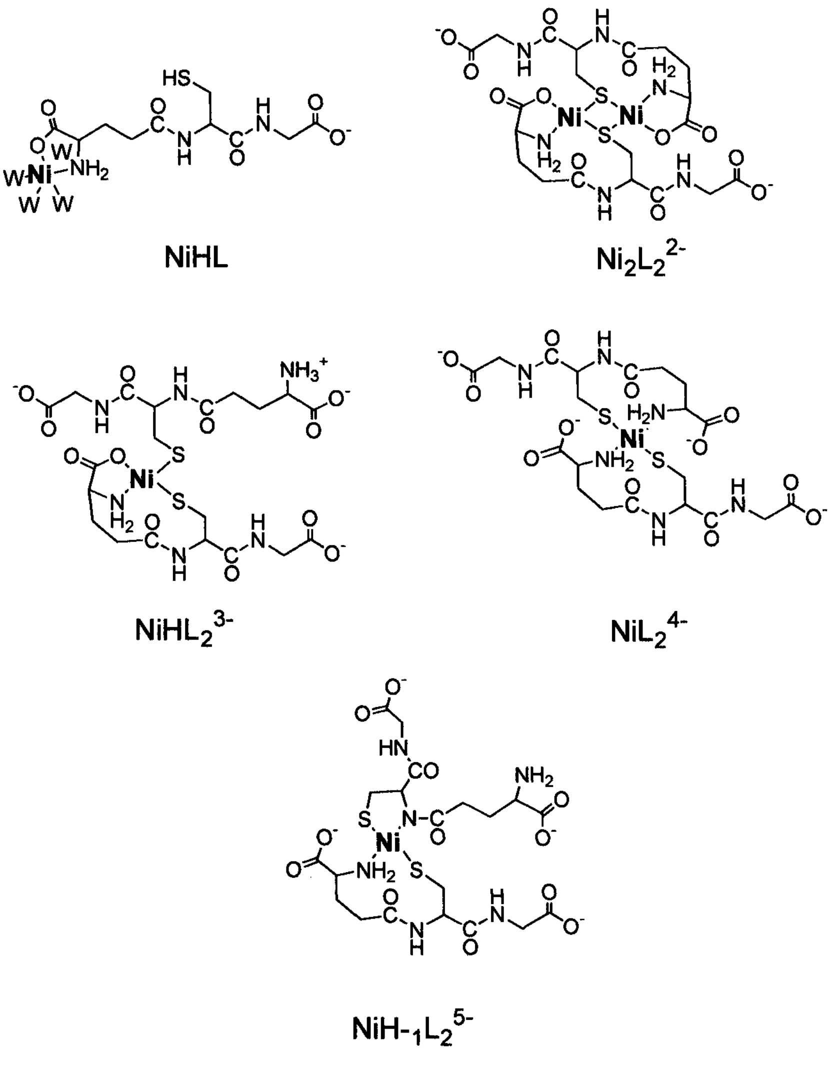

**Scheme 4.** The proposed structures of Ni(II)-GSH complexes. W denotes a water molecule.

### AIR OXIDATION OF GSH AND EFFECT OF Ni(II).

Figure 3 provides the pH profile of the rate constant for air oxidation of uncomplexed GSH to GSSG, demonstrating a perfect match between the rate of this process and the occurrence of the $HL^{3-}$ protonation form of GSH /30/. Our unpublished results indicate that this correlation is caused by the presence of a salt bridge between the protonated $NH_3^+$ and the deprotonated $S^-$ in GSH. The distance between these groups, ca. 2.85 Å, is such that GSH can easily accommodate, by dipolar interactions, and activate dioxygen, and other diatomic molecules. Therefore, the air oxidation of GSH is basically an autocatalytic process, which does not depend on the presence of catalytic amounts of transition metals /31/.

Quite surprisingly, we found out that an acceleration of this process by Ni(II) ions in a phosphate/Tris buffer is weak and limited to the alkaline pH range, up to 4-fold at pH 9; no effect was seen at pH 7.4, in the presence of only minor octahedral Ni(II) complexes. The pH profile, presented in Figure 3, demonstrates that the (predominantly) square-planar $NiHL_2^{3-}$ and $NiL_2^{4-}$ complexes were the most effective ones in facilitating air oxidation of GSH. On the other hand, the strictly square-planar $NiH_{-1}L_2^{5-}$ complex did not oxidize GSH /30/.

### CONSEQUENCES FOR PHYSIOLOGY

The stability constants, obtained for Zn(II) and Ni(II) complexes of GSH, allowed for quantitative estimations of their biological relevance. Intracellular Zn(II) is tightly controlled, at least in part, by proteins, and the estimates, available for *E. Coli*, suggest that the global distribution of Zn(II) is under kinetic rather than thermodynamic control /12/. The situation may be different in eukaryotic cells, where "free" or loosely bound Zn(II) ions have been reported /32, 33/. Nevertheless, a very high variety of zinc proteins, such as hydrolytic enzymes or zinc fingers, and the lack of essential data on them, such as binding constants and number of copies, makes it impossible to even roughly estimate the intracellular Zn(II) speciation. An alternative approach, which can provide a view on the possible involvement of GSH in Zn(II) speciation, is to calculate partitions between GSH (L) at a given physiological concentration and pH on one hand and a range of hypothetical competing ligands (Z), with various binding constants, on another. Such calculations provide a competitivity index of ligand L (or the pair of ligands L, A in the case of ternary complexes) towards a metal ion M, defined as the logarithm of the conditional stability constant of MZ, the metal complex of Z, such that $\Sigma_{ijkl}(/M_iH_jL_kA_l/ = /MZ/$. The value of this index depends on the assumed total concentrations of the metal ion and the competitor Z. The index for GSH, calculated for Zn(II) and Z at 0.2 mM (based on ref. /12/) and pH 7.4 is 8.05 at 10 mM and 6.1 at 1 mM /25/. This means that the range of zinc proteins that may be controlled by GSH includes those with Zn(II) binding constants between $10^6$ and $10^8$. This tentative result indicates that the local concentration of GSH may regulate the activity of some, but not all zinc enzymes and structural proteins /33/. On the other hand, the effect of histidine on the intracellular fate of Zn(II) may be very limited, due to the relatively low binding constant and limited concentration *in vivo* /25/. These results are very preliminary by their very nature and should be treated merely as a guide for designing relevant experiments. One interesting venue may be to study the formation of potential ternary

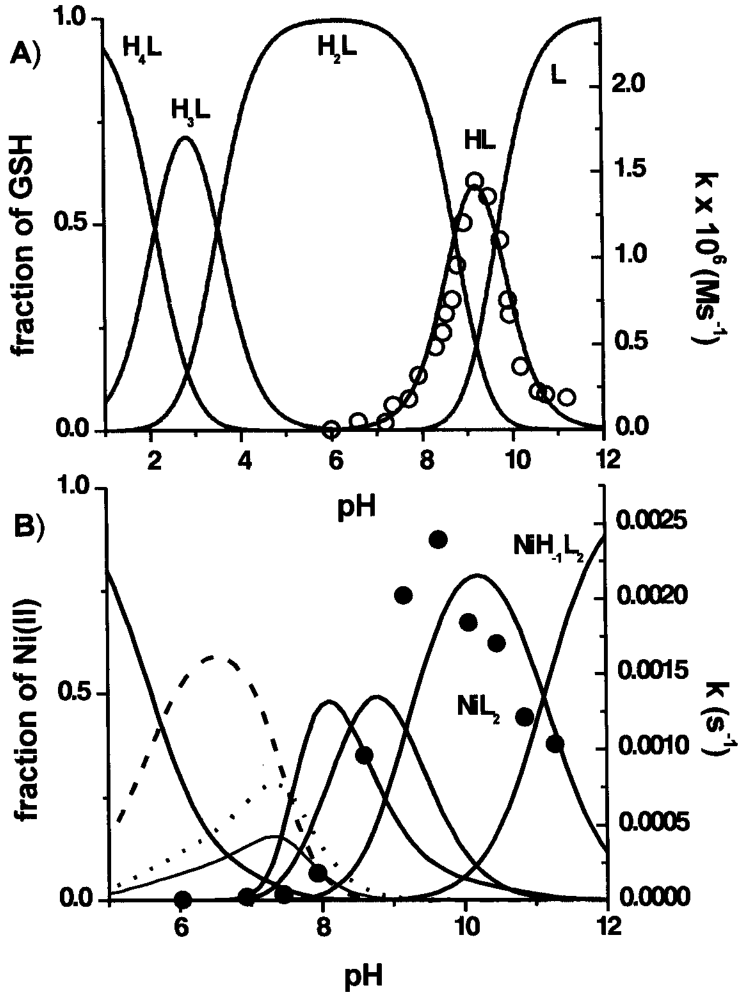

**Fig. 3:** The pH dependence of the kinetic rate constant of air oxidation of GSH to GSSG (initial 2 mM GSH), overlaid on the distribution of protonation macrospecies of GSH (A) and distribution of Ni(II)-GSH complexes (initial 2 mM GSH and 0.5 mM Ni(II)) (B). The dashed and dotted lines represent molar fractions of Ni(II) complexes with phosphate and Tris buffers, respectively. Medium: 50 mM Tris, 50 mM Borax 50 mM $Na_2HPO_4$ and 50 mM $Na_3PO_4$.

Zn(II) complexes with GSH and other chelators, abundant intracellularly, such as nucleotides (*e.g.* ATP).
In contrast with Zn(II), Ni(II) is not physiological in humans and has no dedicated metabolic pathways or transport and storage proteins. Therefore, the attempts to estimate its general speciation and deduce the role of GSH from such calculations are justified /30, 34/. Our recent results indicate that GSH may play only a minor role in Ni(II) speciation, in favor of histidine, ATP and histone proteins. This fact, together with the lack of oxidative activity of Ni(II) towards GSH at pH 7.4, casts doubts on the direct relevance of GSH for Ni(II) toxicity. The indirect effects may, however, be more interesting. We have recently presented the study of Ni(II) assault on the zinc finger of XPA, a crucial protein of the nucleotide excision pathway of DNA repair /35/. The results on GSH complexes of Zn(II) and Ni(II), presented in this review, allow to analyze the effect of increasing GSH concentrations on the equilibrium between Zn(II) and Ni(II) forms of the XPA zinc finger peptide. The results are presented in Figure 4, which clearly demonstrates the synergy between GSH and Ni(II) in depleting the zinc finger peptide of Zn(II). This effect is due to the fact that the stability constants of GSH complexes of Zn(II) are higher from those of Ni(II) (e.g. those for $MHL_2$ and $ML_2$ by 2 orders of magnitude, Table 2), and therefore the increasing GSH concentrations provide a suitable sink for Zn(II).

Overall, our recent results on GSH complexes with Zn(II) and Ni(II) have provided a good basis for predicting interesting physiological effects and may be useful for design and interpretation of suitable biological experiments.

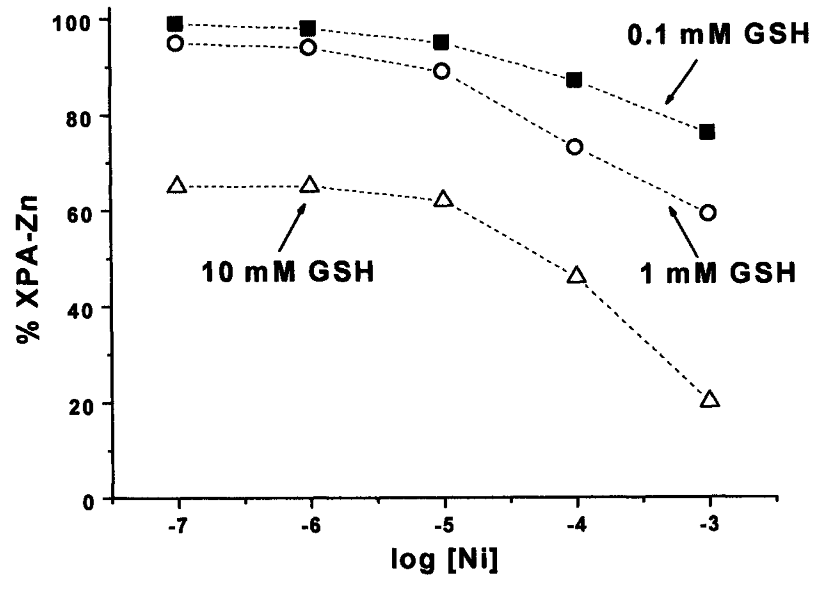

**Fig. 4:** The effect of GSH concentration on a hypothetical competition between Zn(II) and Ni(II) for the binding to XPA zinc finger, based on the data presented in ref. /35/.

**ACKNOWLEDGMENTS**

The authors thank the Polish Committee for Scientific Research (KBN), grant 4 T09A 030 22 for support. A. Krężel thanks the Polish Science Foundation for the travel grant (ISABC 7).

**REFERENCES**

1. A. Meister and M. E. Anderson, *Annu. Rev. Biochem.*, **52**, 711 (1983).
2. D. A. Dickinson and H. J. Forman, *Biochem. Pharmacol.*, **64**, 1019 (2002).
3. H. Sies, *Free Rad. Biol. Med.*, **27**, 916, (1999).
4. W. Maret, *Proc. Natl. Acad. Sci. U.S.A.*, **91**, 237 (1994).
5. L. J. Jiang, W. Maret and B. L. Vallee, *Proc. Natl. Acad. Sci. U.S.A.*, **95**, 3483 (1998).
6. A. Krężel and W. Bal, *Acta Biochim. Polon.*, **46**, 567 (1999).
7. N. Ballatori, *Adv. Pharm.*, **27**, 271 (1994).
8. M. S. Nasir, C. J. Fahrni, D. A. Suhy, K. J. Kolodsik, C. P. Singer and T. V. O'Halloran, *J. Biol. Inorg. Chem.*, **4**, 775 (1994).
9. J. M. Berg and Y. Shi, *Science*, **271**, 1081 (1996).
10. C. Jacob, W. Maret and B. L. Vallee, *Biochem. Biophys. Res. Commun.*, **248**, 569 (1998).
11. L.-J. Jiang, M. Vasak, B. L. Vallee and W. Maret, *Proc. Natl. Acad. Sci. U.S.A.*, **97**, 2503 (2000).
12. C. E. Outten and T. V. O'Halloran, *Science*, **292**, 2488 (2001).
13. D. Atar, P. H. Backx, M. P. Appel, W. D. Gao and E. Marban, *J. Biol. Chem.*, **270**, 2473 (1994).
14. W. Bal and K. S. Kasprzak, *Toxicol. Lett.*, **127**, 55 (2002).
15. W. Li, Y. Zhao and I. N. Chou, *Toxicology*, **77**, 65 (1993).
16. S. Lynn, F. H. Yew, J.-W. Hwang, M.-J. Tseng and K. Y. Jan, *Carcinogenesis*, **15**, 2811 (1994).
17. W. Li, Y. Zhao and I. N. Chou, *Toxicol. Appl. Pharmacol.*, **136**, 101 (1996).
18. K. Miyoshi, Y. Sugiura, K. Ishizu, Y. Iitaka and H. Nakamura, *J. Am. Chem. Soc.*, **102**, 6130 (1980).
19. A. M. Corrie, M. D. Walker and D. R. Williams, *J. Chem. Soc. Dalton Trans.*, 1012 (1976).
20. K. Varnagy, I. Sovago and H. Kozłowski, *Inorg. Chim. Acta*, **151**, 117 (1988).
21. J. Fuhr and D. L. Rabenstein, *J. Am. Chem. Soc.*, **95**, 6944 (1973).
22. W. Bal, A. Krężel, J. Wójcik and M. Maciejczyk, *J. Inorg. Biochem.*, **86**, 135 (2001).
23. M. Gelinsky, R. Vogler and H. Vahrenkamp, *Inorg. Chim. Acta*, **334**, 230 (2003).
24. M. Rombach, M. Gelinsky and H. Vahrenkamp, *Inorg. Chim. Acta*, **334**, 25 (2002).
25. A. Krężel, J. Wójcik, M. Maciejczyk and W. Bal, *Chem. Commun.*, 704 (2003).
26. A. Krężel and W. Bal, *Toxicol. Lett.*, **123** (Suppl. 1), 50 (2001).
27. G. Formicka-Kozlowska, P. M. May and R. Williams, *Inorg. Chim. Acta*, **46**, L51 (1980);
28. I. Sóvágó and R. B. Martin, *J. Inorg. Nucl. Chem.*, **43**, 425 (1981).
29. B. Jeżowska-Trzebiatowska, G. Formicka-Kozłowska and H. Kozłowski, *Chem. Phys. Lett.*, **42**, 242 (1976).

30. A. Krężel, W. Szczepanik, M. Sokołowska, M. Jeżowska-Bojczuk and W. Bal, *Chem. Res. Toxicol.*, **16**, 855 (2003).
31. A. Krężel and W. Bal (unpublished data).
32. J. Benters, U. Flögel, T. Schäfer, D. Leibfritz, S. Hechtenberg and D. Beyersmann, *Biochem. J.*, **322**, 73 (1997).
33. S. R. Kar, A. C. Adams, J. Lebowitz, K. B. Taylor and L. M. Hall, *Biochemistry*, **36**, 15343 (1997).
34. W. Bal, H. Kozłowski and K. S. Kasprzak, *J. Inorg. Biochem.*, **79**, 213 (2000).
35. W. Bal, T. Schwerdtle and A. Hartwig, *Chem. Res. Toxicol.*, **16**, 242 (2003).

